# Benchmarking ML in ADMET predictions: the practical impact of feature representations in ligand-based models

**DOI:** 10.1186/s13321-025-01041-0

**Published:** 2025-07-21

**Authors:** Gintautas Kamuntavičius, Tanya Paquet, Orestis Bastas, Dainius Šalkauskas, Alvaro Prat, Hisham Abdel Aty, Aurimas Pabrinkis, Povilas Norvaišas, Roy Tal

**Affiliations:** AI Chemistry, Ro5, 2801 Gateway Drive, 75063 Irving, TX USA

## Abstract

This study, focusing on predicting Absorption, Distribution, Metabolism, Excretion, and Toxicology (ADMET) properties, addresses the key challenges of ML models trained using ligand-based representations. We propose a structured approach to data feature selection, taking a step beyond the conventional practice of combining different representations without systematic reasoning. Additionally, we enhance model evaluation methods by integrating cross-validation with statistical hypothesis testing, adding a layer of reliability to the model assessments. Our final evaluations include a practical scenario, where models trained on one source of data are evaluated on a different one. This approach aims to bolster the reliability of ADMET predictions, providing more dependable and informative model evaluations.

**Scientific contribution**

This study provided a structured approach to feature selection. We improve model evaluation by combining cross-validation with statistical hypothesis testing, making results more reliable. The methodology used in our study can be generalized beyond feature selection, boosting the confidence in selected models which is crucial in a noisy domain such as the ADMET prediction tasks. Additionally, we assess how well models trained on one dataset perform on another, offering practical insights for using external data in drug discovery.

## Introduction

The Absorption, Distribution, Metabolism, Excretion, and Toxicology (ADMET) of compounds are commonly estimated throughout drug discovery projects, as the feasibility of a compound to become a viable drug highly depends on it. Through the years, a lot of work has gone into building and evaluating machine learning (ML) systems designed to predict molecular properties that are associated with ADMET. Public curated datasets and benchmarks for ADMET associated properties are becoming increasingly available to the community, creating the opportunity for more widespread exploration of ML algorithms and techniques in this space. The Therapeutics Data Commons (TDC) ADMET leaderboard showcases this [1], highlighting a wide variety of models, features, and processing methods investigated over the past two years.

The studies showcased on the leaderboard often focus on comparing different ML models and architectures, whereas the selection of compound representations is either not justified, or analyzed with limited scope. For instance, many approaches concatenate a number of compound representations at the onset for the assessment of various models. While compound representation and feature selection justification is lacking, these approaches often yield very good results against the TDC benchmarks [2–4]. In the present study, we aim to improve the understanding of the impact of feature concatenation, taking a step further to provide a process that can inform dataset-specific, statistically significant compound representation choices.

Different deep neural network (DNN) compound representations became prevalent over the past years [5–8]. We investigate how the DNN compound representations compare to the more classical descriptors and fingerprints in the ADMET ML domain.

In the present study, we conduct experiments to enlighten the following research questions:

- Which types of algorithms and compound representations are generally suitable for ligand-based machine learning in the ADMET domain?
- Can cross-validation hypothesis testing serve as a more robust model comparison than a hold-out test set in the ADMET domain?
- How important are various forms of model optimization in a practical scenario?
- What is the impact on the model performance when available external data of the same property is used in combination with internal data?

Public ADMET datasets are often criticised with regards to data cleanliness. Issues range from inconsistent SMILES representations and multiple organic compounds found in a single fragmented SMILES string, to duplicate measurements with varying values and inconsistent binary labels. Different binary labels for the same SMILES string have also been observed across train and test sets. In order to mitigate these issues in the present study, we begin by applying a set of data cleaning procedures. The data cleaning results in the removal of a number of compounds across datasets^1^.

At a high level, the experiments in this study are carried out sequentially, achieving the following:

1. A model architecture is chosen to use as a baseline as well as optimize in further experiments;
2. Features are combined iteratively until the best-performing combinations are identified;
3. Hyperparameters of the chosen model architecture are tuned in a dataset-specific manner;
4. Cross-validation hypothesis testing is done in order to assess the statistical significance of the optimization steps;
5. Test set performance is evaluated, assessing the impact of the previous optimization steps, as well as the contrast between the hypothesis test outcomes and test set changes;
6. The optimized models are evaluated in a practical scenario, where models trained on one data source are evaluated on a test set from a different source, for the same property and;
7. Finally, the optimized model is trained on a combination of data from two different sources, to mimic the scenario when external data is combined with increasing amounts of internal data.

## Related work

Fang *et al*. have assessed the performance of many popular ML models on their internal ADME assay data [9]. While they look at a variety of models, the investigation of different compound representations are more limited, exploring only combinations of RDKit descriptors and functional connectivity fingerprints with a radius of 4 (FCFP4). Their experiments were done in a sequential manner and evaluations were carried out on temporal splits. They have also shared their in-house ADME assay results for  around 3000 purchasable compounds. This dataset has been invaluable in our study, allowing us to assess the impact of external data on internal data prediction.

Most recently, Green *et al*. carried out a study on ADMET and quantitative structure-activity relationship (QSAR) tasks, focusing on a wide range of models and features [10]. They propose a principled implementation of uncertainty estimation (estimates for both aleatoric and epistemic uncertainty) as well as calibration, highlighting the superior performance of Gaussian Process (GP) based models in particular. While the group showed GP models to consistently perform the best in bioactivity assays, there was no such clear conclusion for ADMET datasets, for which they have found the optimal model and feature choices to be highly dataset-dependent.

Deng et al. have carried out a study [11] that has many similarities with ours; in particular, a number of models were trained with both classical and deep-learned feature representations on ADMET as well as other datasets. The study contains a very thorough analysis and comparison of generally popular ML methods in the area. The authors investigate fixed versus learned (i.e. fine-tuned to the particular dataset) representations, arriving at a conclusion that fixed representations generally outperform learned ones. Moreover, the random forest model architecture was found to be the generally best performing one. In our study we investigate fixed representations, taking a step further to investigate combinations of various representations as well as identify a different best-performing model architecture.

Lane et al. [12] conducted a comprehensive evaluation of multiple classical machine learning methods and deep learning architectures across a vast array of bioactivity datasets extracted from ChEMBL. Their rigorous benchmarking employed extensive hyperparameter optimization, enabling fair comparisons among algorithms including Random Forests, Support Vector Machines, k-Nearest Neighbors, AdaBoost, Nave Bayes, and deep neural networks. By systematically tuning hyperparameters for each model, they provided clear evidence of the relative performance of diverse machine learning techniques in molecular property prediction tasks. In contrast, our study prioritizes an exploration of diverse molecular feature combinations across a wide range of ADMET datasets. Our work complements the approach of Lane et al. by addressing the aspect of feature representation alongside their detailed algorithmic evaluation.

## Methods

### Data

#### Datasets

Datasets pertaining to the ADMET properties of small molecules were obtained from different public sources as laid out in Table 1.

From TDC [1], the single_pred method was used to obtain exclusively human ppbr_az data. For all other TDC datasets, the recommended benchmark_group method and scaffold splits were used. Kinetic solubility from the National Institute of Health (NIH) as described by Guha *et. al* was obtained from PubChem [13]. Biogen published in vitro ADME experiments for a set of non-proprietary small-molecule compounds and made them publicly available [9]. The scaffold split method within the DeepChem library was used to split the Biogen and NIH datasets.

#### Data cleaning

Data cleaning was aimed at getting consistent SMILES representations, and to remove noise due to measurement ambiguity. For in vitro and in vivo assays, we assume that the compound, or salt thereof, is soluble in the medium used, and that the effect observed can be attributed to the parent organic compound in the case of salts.

For solubility, the properties of different salts of the same compound may differ depending on the salt component. All records pertaining to salt complexes were removed from solubility datasets.

The standardisation tool by Atkinson *et al*. were used to clean the compound SMILES strings [14]. We included two modifications to definitions within the tool.

- Boron and silicon were added to the list of organic elements as such that an organic compounds is defined as a compound that only consists of the following elements: H^1^, C^6^, N^7^, O^8^, F^9^, P^15^, S^16^, Cl^17^, Br^35^, I^53^, B^5^, and Si^14^.
- Positive and negative hydrogen ions were added to the pre-defined salt list as they were present as salt components in some datasets, e.g. as [H+].[Cl-].

In addition, a truncated salt list was created to omit salt components that can in themselves be a parent organic compound with a property measurement e.g. citrate/citric acid. The truncated list was created by excluding components that contain two or more carbons from the tool’s pre-defined list. 36 components were excluded as such.

The following steps were the taken to conduct data cleaning across the datasets:

- Remove inorganic salts and organometallic compounds from the datasets.
- Extract organic parent compounds form their salts forms.
- Adjust tautomers to have consistent functional group representation.
- Canonicalize SMILES strings.
- De-duplication. We either keep the first entry if the target values of the duplicates are consistent, or remove the entire group if they are inconsistent. "Consistent" is defined as exactly the same for binary tasks (i.e. the target values of the group are either all 0 or all 1), and within 20% of the inter-quartile range for regression tasks.

Finally, since the datasets are relatively small, visual inspection of the resultant clean datasets were done using DataWarrior [15].

Many of the ADMET endpoints in the datasets are log-transformed. To address highly skewed distributions, we transformed another three of the TDC datasets namely, clearance_microsome_az, half_life_obach and vdss_lombardo. For these, the metrics shown in this work are computed on the log transformed values instead of the original ones listed in Table 1.

**Table 1:** Dataset descriptions

Dataset name (Table)	Property	Units	Size (distribution)
TDCommons–regression			**(IQR)**
caco2_wang (Table 22)	Cell effective permeability	Log(Papp)	632 (− 5.7; − 4.6)
lipophilicity (Table 42)	Octanol/water distribution	LogD	4199 (− 0.6; 0.8)
ppbr_az (Table 37)	Human plasma protein binding	% bound	1614 (85; 99)
ld50_zhu (Table 23)	50% lethal dose	log(kg$\cdot$mol$^{-1}$)	7308 (1.9; 3.0)
vdss_lombardo (Table 36)	Steady-state volume of distribution	L$\cdot$kg$^{-1}$	1105 (0.3; 2.8)
half_life_obach (Table 29)	Terminal phase half life from IV administration	hr	663 (1.8; 11)
clearance_microsome_az (A28)	Human liver microsome intrinsic clearance	mL$\cdot \text {min}^{-1}g^{-1}$	1102 (3.0; 43)
TDCommons - binary		**Positive threshold**	**(# positive)**
bioavailability_ma (Table 39)	Human oral bioavailability	F $\ge$ 20%	638 (491)
hia_hou (Table 41)	Human intestinal absorption	FA > 30%	578 (500)
pgp_broccatelli (Table 40)	P-glycoprotein inhibition	IC$_{50}$ < 15 $\mu$M, or > 25% inhibition relative to control	1212 (647)
bbb_martins (Table 38)	Blood-brain barrier penetration	logBB $\ge$ -1	1945 (1490)
cyp2c9_veith (Table 33)	CYP2C9 inhibition	inhibition below 57 μM	11,927 (3993)
cyp2d6_veith (Table 35)	CYP2D6 inhibition	inhibition below 57 μM	12,960 (2491)
cyp3a4_veith (Table 34)	CYP3A4 inhibition	inhibition below 57 μM	12,170 (5037)
cyp2c9_substrate_carbonmangels (Table 30)	CYP2C9 metabolism	As per literature annotation [16]	666 (141)
cyp2d6_substrate_carbonmangels (Table 32)	CYP2D6 metabolism	As per literature annotation [16]	663 (190)
cyp3a4_substrate _carbonmangels (Table 31)	CYP3A4 metabolism	”	667 (354)
herg (Table 26)	hERG inhibition	pIC$_{50}$ $\ge$ 4.4	603 (414)
ames (Table 25)	Mutagenicity (bacterial reverse mutation assay)	Colony growth in at least 1 of 5 strains	7220 (3941)
dili (Table 24)	Drug-induced liver injury (hepatotoxicity)	According to FDA approved labeling [17]	466 (230)
**NIH-regression**			**(IQR)**
Solubility (Table 27)	Kinetic aqueous solubility	$\mu$g$\cdot$mL	36,238 (4.0; 35)
**Biogen-regression**			**(IQR)**
hppb	Human plasma protein binding	log(% unbound)	194 (0.2; 1.5)
rlm (Table 18)	Rat liver microsomes intrinsic clearance	log(mL$\cdot$min$^{-1}\cdot$kg$^{-1}$)	3054 (1.7; 2.8)
solubility (Table 19)	Kinetic aqueous solubility	log(μg$\cdot$mL) (pH 6.8)	2173 (1.2; 1.7)
hlm (Table 21)	Human liver microsome intrinsic clearance	log(mL$\cdot$min$^{-1}\cdot$kg$^{-1}$)	3087 (0.7; 1.8)
mdr1-mdck (Table 20)	P-Glycoprotein efflux ratio	log(B-A/A-B)	2642 (− 0.2; 0.9)

### Modeling and evaluation

#### Models

The machine learning algorithms included in the present study range from classical models to more recent neural networks. Included is Support Vector Machines (SVM) [18], tree-based methods comprising Random Forests (RF) [19] and gradient boosting frameworks LightGBM [20] and CatBoost [21], as well as Message Passing Neural Networks (MPNN) as implemented by Chemprop [22] (Ver. 1.6.1).

#### Features

Various descriptors, fingerprints, and embeddings were used on their own or in combination. The following descriptors and fingerprints were implemented using the RDKit cheminformatics toolkit [23]: RDKit descriptors (rdkit_desc), Morgan fingerprints with a radius of 2 (ecfp4) [24], atom pair fingerprints (atom_pair) [25], Avalon Fingerprints (avalon) [26], and Extended reduced graph (erg) descriptors [27]. Mordred fingerprints (mordred) were obtained using the Mordred molecular descriptor calculator [28]. Embeddings used includes Mol2vec (mol2vec) [8], Graph Representation frOm self-supervised mEssage passing tRansformer (grover) [7], MolFormer (molformer) [6], and BARTSMILES (bartsmiles) [5]. For MolFormer, we made use of the open-source model version that was trained on 10% of the data. BARTSMILES embeddings were extracted through loading the fairseq Bart model with the pre-trained BARTSMILES checkpoint, encoding the SMILES and extracting their features using fairseq’s functions, and averaging them to create a vector for each datapoint. MegaMolBart representations were computed using Nvidia’s BioNemo API [29].

#### Evaluation metrics

Following the approach of Green *et al.*, we employed normalized root-mean-square error (NRMSE) as our primary evaluation metric for regression tasks [10]. NRMSE is computed by dividing the standard RMSE by the interquartile range (IQR) of the training set. This normalization provides a dimensionless measure, facilitating meaningful comparisons across datasets with widely varying scales and ranges (note in Table 1 we observe the IQR of e.g. ppbr_az is of size 14, whereas ld50_zhu is 1.1). While metrics such as the coefficient of determination (R2) are widely used and are indeed utilized for supplementary comparative analyses (see Fig. 9), NRMSE serves as our primary metric because it explicitly normalizes error scales, making it particularly suitable for direct comparisons across diverse experimental conditions. Moreover, as our study focuses on a nonlinear model (catboost), it is more appropriate to discuss the results using NRMSE to avoid confusion.

For binary classification tasks, we selected the area under the precision-recall curve (AU-PRC) as the evaluation metric, given the occasional class imbalance present in our datasets (consider e.g. bioavailability_ma and cyp2c9_substrate_carbonmangels in Table 1). AU-PRC emphasizes the performance of models in correctly identifying the minority class and is less influenced by class imbalance compared to metrics such as the area under the receiver operating characteristic curve (ROC-AUC). While ROC-AUC is widely used [30] and also used in our study for comparative analysis (see Fig. 10), it tends to present overly optimistic performance estimates in highly imbalanced scenarios by equally weighting true negatives, which dominate the evaluation, alongside true positives. In contrast, AU-PRC specifically accounts for the precision-recall trade-off, offering a clearer representation of model effectiveness in identifying the less frequent positive outcomes [31, 32]. Although standard metrics such as accuracy, precision, recall, and the F1-score are common alternatives [30, 33], they are threshold-dependent and require choosing a specific operating point for model evaluation. Such metrics can provide misleading results depending on the selected threshold and do not provide a comprehensive assessment of performance across all possible thresholds. Hence, we opted for AU-PRC, which provides an integrated, threshold-independent evaluation, delivering a robust measure of performance suitable for our study.

#### Statistics

The Friedman $\chi ^2$ test [34] was used to detect differences across multiple pairwise model and feature comparisons. The test compares ranks rather than actual values, making it appropriate for non-normally distributed data.

1 $$\begin{aligned} \chi ^2_F = \frac{12N}{k(k+1)} \left[ \sum _{j=1}^{k} R_j^2 - \frac{k(k+1)^2}{4} \right] \end{aligned}$$

In Equation 1, in the context of our study, N is the number of pairwise comparisons (e.g. all 130 model and dataset pairs that use rdkit_desc features), k is the number of methods (e.g. the 10 different feature sets), and $R_j$ is the total sum of the ranks of the method in question (e.g the rdkit_desc features).

After significant results were obtained from the Friedman $\chi ^2$ test, we used the Nemenyi post-hoc test [35] to compare multiple groups.

2 $$\begin{aligned} Q_{ij} = \frac{|R_i - R_j|}{\sqrt{\frac{k(k+1)}{6n}}} \end{aligned}$$

The Nemenyi test were chosen due to its robustness to the multiple comparisons problem compared to other methods, such as the Wilcoxon rank sum test [36]. This is because the number of comparisons *k* is taken into account when computing the test statistic $Q_{ij}$ (Eq. 2), which follows the studentized range distribution [37].

The non-parametric nature of the statistical test allowed us to use information from both the binary classification and regression tasks, as only the relative rankings are considered. Therefore, we combined the rankings of the NRMSE values for regression tasks with the rankings of the AU-PRC values for binary classification tasks to perform the post-hoc tests.

### Experiments

The experiments carried out in this study were sequential, each making use of the findings of the previous experiments. They were structured as follows:

1. **Model selection** Initially, we make use of the single-fold experiment setup: train on training set, evaluate of validation set, test set not used. In the first stage, we aim to identify a single model out of the five that we will take forward in more detailed feature combination as well as hyperparameter tuning experiments (default hyperparameters of each model were used in this step; these are outlined in Tables 12, 13, 14, 15, 16, 17). A model is trained for each dataset, feature, and model combination, and the models are ranked (1 to 5) in comparison to each other. This results in $25 \cdot 11 \cdot 5 = 1375$ trained models. Subsequently, we also compare models trained using each of the features (except mordred)^2^ in combination with rdkit_desc, a popular feature representation that we have also found to work well. The performance of a model trained on a combination of features is important as it can suggest whether the model architecture will continue performing well as we expand the number of combined features. This results in another 9 feature combinations, corresponding to $25 \cdot 9 \cdot 5 = 1125$ trained models. The models are again ranked (1 to 5), their average rankings are investigated and the significance of the best models’ performance is assessed using the Nemenyi test.
  Note, however, that hyperparameters were not optimized at this stage. While in principle, hyperparameter optimization should be performed to compare architectures, in the current study we accepted default parameters due to the large number of models constructed and the limitations of computational resources, instead prioritizing the investigation of feature combinations. For more principled model evaluations, see [12, 38–40].
2. **Feature combination** Once the model is selected, the next step is to understand the impact of using multiple feature representations on the efficacy of ADMET models. In order to assess this, we iteratively add features one-by-one, starting with the best-performing rdkit_desc, evaluating the change in performance across the datasets at every step. Features are added until we observe no improvement from the additional features, thus avoiding the inclusion of noise into the training process. In these experiments, we discovered that the impact of feature choice is very different for regression and binary classification datasets. We therefore perform this iterative feature addition separately for regression and binary classification datasets. Once final feature combinations are selected, hyperparameter optimization is done.
3. **Hyperparameter optimization** The hyperparameter optimization is performed in a dataset-specific manner, unlike the previous optimization steps which were evaluated across all the datasets at once. We use a simple 3-fold cross-validation method, using random search with 20 iteration steps. Random search was chosen due to its robustness compared to grid search when redundant hyperparameters might be involved. In particular, in the presence of unimportant hyperparameters, the efficiency (per unit compute) of a grid search decreases exponentially while random search stays constant, with respect to the exploration of important hyperparameters. This lets us investigate a large range of hyperparameters without worrying that the space will be poorly explored. This type of reasoning is in line with the findings from Bergstra et al. [41].
  The hyperparameter grid is defined in Table 2, chosen based on recommendations from the model authors [21].
4. **Cross-validation hypothesis testing** To understand whether the optimization choices made in previous steps are meaningful, we perform large scale cross-validation hypothesis testing. In order to maximize the statistical power of the Nemenyi test, we use 10 cross-validation folds. Four model configurations are evaluated:
  Baseline: catboost with default parameters and rdkit_desc feature representations.
  catboost with default parameters and rdkit_desc + ecfp4 feature representation combination, which is a popular choice in literature
  catboost with default parameters and the optimized features, as described in Section 4.2.
  catboost with optimized hyperparameters as well as optimized features.
  For each of the four model configuration, we train a model on 25 datasets with 10 folds each, resulting in 250 sets of four paired evaluations to draw the statistical test from.
5. **Test set evaluation** Finally, the baseline as well as optimized models are evaluated on the held-out test set and the improvements are listed for each dataset. Importantly, the performances are shown per-dataset, and they are compared parametrically (NRMSE for regression tasks and AU-PRC for binary classification); in the previous analyses we have used relative ranking to identify the superior models, without considering the magnitude of the improvement. Moreover, together with test set results we observe the hypothesis test results for each dataset from the previous 10-fold CV experiment. The hypothesis test outcome is compared to the change in test set performance.
6. **Transferability** Properties for which two or more datasets were obtained were used to investigate whether, for a given property, data from one laboratory can be used to predict the measurements from a different laboratory. For these transferability investigations across datasets of the same property, the respective dataset values were transformed to align the measurement units, and overlapping compounds were removed, as well as those with values greater or less than a threshold. The three properties, human plasma protein binding (hPPB), human liver microsome intrinsic clearance (HLM), and kinetic solubility (solubility), for which there are both a Biogen dataset as well as a dataset from TDC (AstraZeneca) or the NIH, were used. For each property, two sets of experiments were conducted.
  Firstly, the optimized models from the previous experiments are evaluated in a practical scenario: catboost models were trained on either the TDC or NIH data, and tested on Biogen data. This was done using all four model configurations described in the cross-validation experiment.
  Secondly, an experiment is conducted to assess the impact of using external (hypothetically represented by TDC or NIH) together with internal (hypothetically represented by Biogen) data. 50% of the Biogen data were isolated as a test set via a scaffold split. The remaining 50% of the Biogen data was incrementally (either 0.5% or 5% increments up to 50%) added to the additional dataset’s data, or used for finetuning or data adaptation thereof. Both unsupervised (KMM, KLIEP, or NearestNeighborsWeighting) and supervised (TrAdaBoostR2) adaptation methods were considered during experimentation. Where data were combined, the resulting training sets contained increasing proportions of Biogen data. Compositions of maximally combined datasets varied across the three properties and are summarised in Table 3. catboost models with the optimal hyperparameters for the property were constructed with these training sets using the combined features. The models were evaluated based on RMSE against the isolated test set. Models were also trained using only the Biogen portions of the training data to asses the change in performance due to the additional data. The experiments were repeated with 5 different 50% scaffold splits of the Biogen datasets.

**Table 2:** CatBoost hyperparameter grid used in the random search with 20 iterations

Hyperparameter	Values^a,b^
depth	4, **6**, 8, 10
learning_rate	0.01, **0.03**, 0.05, 0.1, 0.3
iterations	500, **1000**, 2000
l2_leaf_reg	1, **3**, 5, 7
bagging_temperature	0, **1**, 3, 5

^a^The total size of the grid amounts to 960 possible combinations

^b^The default hyperparameter values are bolded

## Results

### Model selection

Table 4 shows the relative model performances across the single and combined feature experiments. While catboost and svm have comparable average rankings in the single feature experiment, catboost comes out superior once the features are combined with rdkit_desc.

**Fig. 1:**
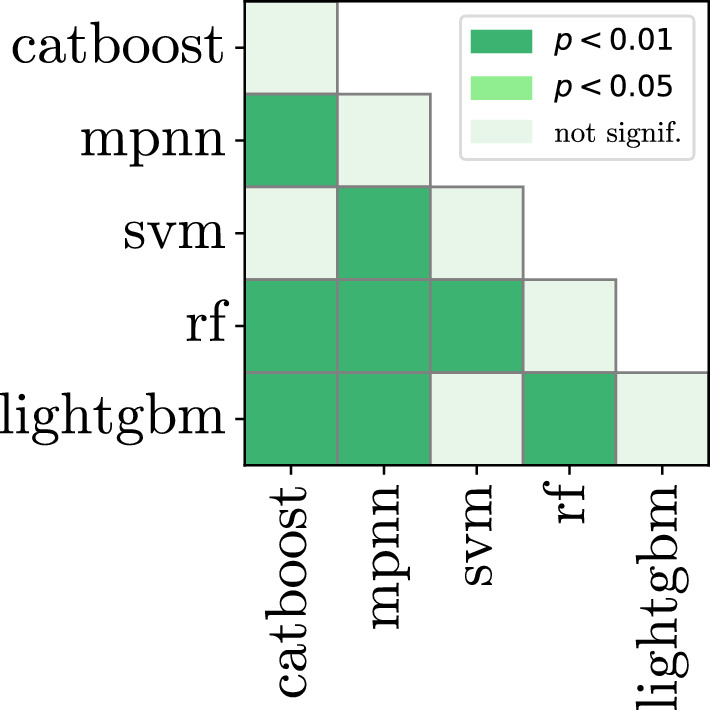
Model comparison, p-value heatmap according to the Nemenyi test of all 11 features trained individually. Both axes represent each model type. Each model is trained 275 times, for every dataset and feature combination. Average ranks of each model are shown in Table 4

**Fig. 2:**
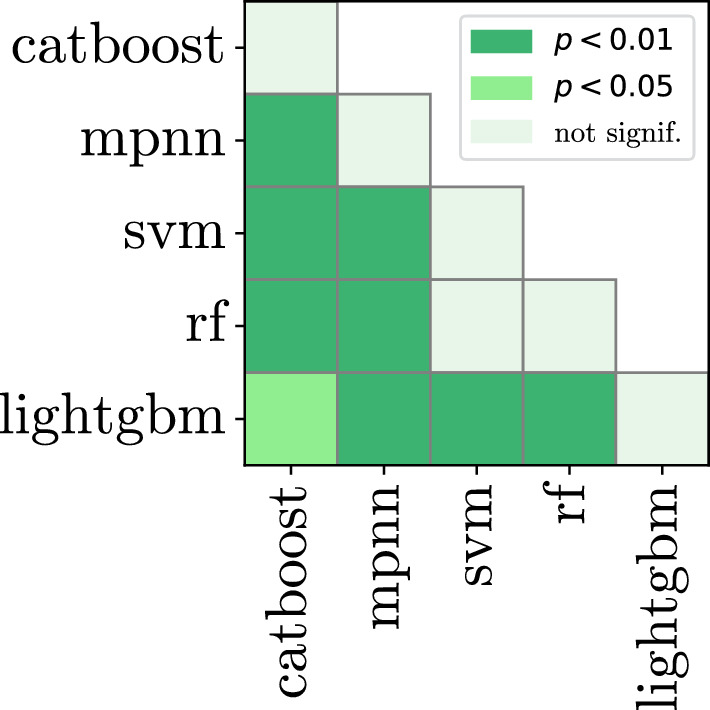
Model comparison, p-value heatmap according to the Nemenyi test of 9 features (previous 11 minus rdkit_desc and mordred) trained in combination with rdkit_desc. Both axes represent each model type. Each model is trained 225 times, for every dataset and feature combination. Average ranks of each model are shown in Table 4

This is corroborated by the p-value heatmaps (Figs. 1, 2). We observe that in the single feature experiment, catboost is not significantly different from svm and lightgbm. However, the difference becomes significant when the combination of two feature sets are used.

Based on these findings, in the subsequent experiments we chose to use the catboost model, both for investigating feature combinations and hyperparameter optimization.

**Table 3:** Maximum combined dataset compositions for transferability experiments

Property	Maximum combined dataset^a^	Fraction Biogen data^b^	Biogen test set size
hPPB	1704	0.053	92
HLM	1781	0.590	1059
solubility	37,308	0.029	1076

^a^TDC or NIH data combined with 50% of the Biogen data

^b^Fraction of the maximum combined dataset represented by the Biogen data

**Table 4:** Average model rankings in the single-fold experiment

Model	Average rank, single feature^a,c^	Average rank, two features^b,c^
catboost	**2.36**	**2.07**
mpnn	4.03	4.28
svm	**2.52**	3.01
rf	3.29	3.13
lightgbm	2.79	2.52

^a^In the single-feature experiment, all 11 features are all trained, resulting in 1325 total models trained across all datasets

^b^For the two features experiment, rdkit_desc features are concatenated with the other features (except mordred), resulting in 1125 total models

^c^The rankings are relative model performances based on the validation set. Corresponding p-value heatmaps can be seen in Figs. 1, 2

### Feature combination

First, we evaluate the catboost models trained on a single feature representation, confirming rdkit_desc as the superior standalone feature representation in ADMET tasks. The average ranks of each feature across the datasets are shown in Table 5. We can see that its superiority is more prominent in regression tasks compared to binary classification.

The process of iteratively adding features is shown in Table 6 for regression datasets and 7 for binary classification datasets. We find that the rdkit_desc + erg + ecfp4 + avalon combination performs best on average for regression datasets, and rdkit_desc + erg + avalon for binary classification datasets. Adding more features to these combinations decreases the model performance across the datasets.

**Table 5:** Feature performance comparison in the single-fold evaluation experiment, using a catboost model with default hyperparameters

Feature	Regression^b^	Binary^b^	Overall^a^
molformer	6.91	6.00	6.38
bartsmiles	8.91	6.85	7.83
grover	5.91	6.15	6.08
mol2vec	5.82	6.23	6.08
atom_pair	7.09	6.62	6.79
ecfp4	7.91	6.38	7.04
rdkit_desc	**1.91**	**4.31**	**3.21**
erg	7.45	9.08	8.33
avalon	7.18	5.69	6.42
mordred	2.45	**4.23**	3.38
megamolbart	4.45	**4.46**	4.46

^a^A model using a particular feature representation is trained 25 times for every dataset, and relative average rankings are shown in the table

^b^Separate rankings are also shown for regression and binary classification datasets, of which there are 12 and 13, respectively

**Table 6:** Performance (average rank) of iteratively added feature representations in **regression** datasets

	rdkit_desc^a^	rdkit_desc	rdkit_desc	rdkit_desc
		+ erg^b^	+ erg	+ erg
			+ ecfp4	+ ecfp4
				+ avalon
No added feature	5.0	3.18	3.18	**1.55**^c^
+ erg	**2.5**	−	−	−
+ ecfp4	3.08	**2.45**	−	−
+ avalon	3.5	2.55	**2.27**	−
+ atom_pair	4.42	4.0	2.55	2.73
+ bartsmiles	9.25	8.0	7.18	6.0
+ grover	7.33	7.18	6.27	5.55
+ megamolbart	5.83	5.45	4.64	4.27
+ mol2vec	5.67	4.82	3.64	2.55
+ molformer	8.42	7.36	6.27	5.36

^a^Each set of base features, denoted in the top row, is combined with an extra feature and their performances are ranked compared to each other within same column

^b^For example, in the first step, the rdkit_desc + erg has the highest average ranking compared to other two feature combinations as well as the baseline rdkit_desc; therefore we set it as the base two-feature combination in the subsequent column where a third feature is added in the same manner

^c^This process continues until adding an extra feature does not show improvement

**Table 7:** Performance (average rank) of iteratively added feature representations in **binary classification** datasets

	rdkit_desc^a^	rdkit_desc + avalon^b^	rdkit_desc + erg + avalon
No extra feature	6.46	4.92	**3.46**^c^
+ avalon	**4.08**	-	-
+ erg	5.38	**3.77**	-
+ atom_pair	5.92	4.69	4.08
+ ecfp4	4.62	4.38	4.46
+ megamolbart	4.38	4.46	4.69
+ bartsmiles	6.62	6.08	5.77
+ grover	6.38	6.38	5.62
+ mol2vec	5.54	5.15	3.62
+ molformer	5.62	5.15	4.31

^a^Each set of base features, denoted in the top row, is combined with an extra feature and their performances are ranked compared to each other within same column

^b^For example, in the first step, the rdkit_desc + avalon has the highest average ranking compared to other two feature combinations as well as the baseline rdkit_desc; therefore we set it as the base two-feature combination in the subsequent column where a third feature is added in the same manner

^c^This process continues until adding an extra feature does not show improvement

### Cross-validation hypothesis testing

**Fig. 3:**
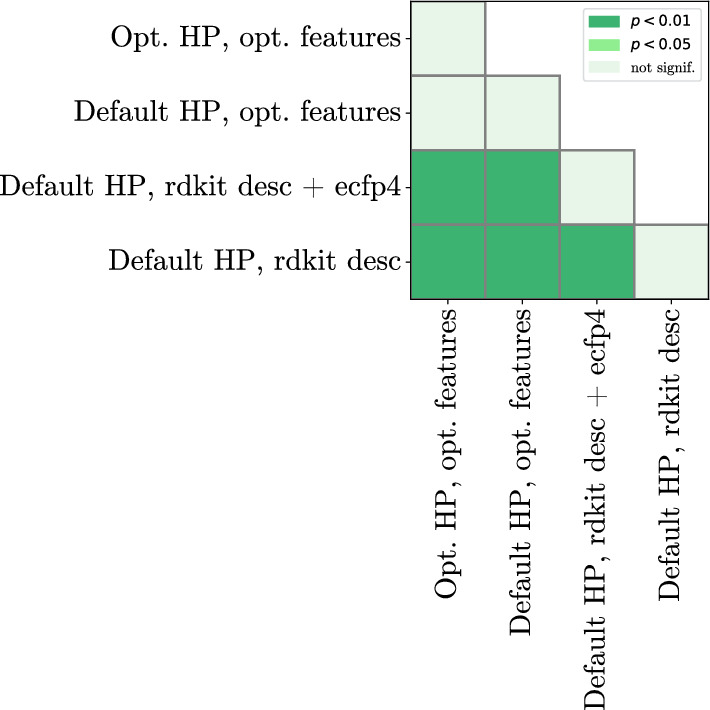
CatBoost model configuration comparison, p-value heatmap according to the Nemenyi test comparing four differently optimized models. Both axes represent each model configuration. Each model configuration is trained 250 times, for each of the 10 folds across 25 datasets. Average rankings of each model configuration are shown in Table 8

The four chosen model configurations are now trained in a 10-fold cross-validation setting, where the large number of paired performance measurements will allow for a thorough assessment of impact of various optimization steps. We can see the average rankings of each model configuration in Table 8. To create this table, 25 (number of datasets) * 10 (number of CV folds) = 250 sets of four model configurations were compared, resulting in high statistical power of the experiment; the results of the corresponding Nemenyi hypothesis tests are shown in Fig. 3.

However, we can also perform these hypothesis tests for every dataset, using only 10 paired measurements for every model configuration. The results of these dataset-specific hypothesis tests (specifically, whether the fully optimized catboost model gives significantly different predictions to the baseline) are shown together with the results on the test set in Table 9.

**Table 8:** Performance of four different CatBoost model configurations, with either default or optimized hyperparameters as well as various feature representation combinations

Features	Model	Average rank^a,b^
rdkit_	catboost	
desc	default	3.09
rdkit +	catboost	
ecfp4	default	2.84
optimized	catboost	
features	default	2.20
optimized	catboost	
features	optimized	**1.84**
	params	

^a^Each configuration is trained across 25 datasets with 10 folds each, and the configurations’ average ranking is reported

^b^The significance of model improvements is visualized in terms of the p-values in Fig. 3

### Test set evaluation

**Fig. 4:**
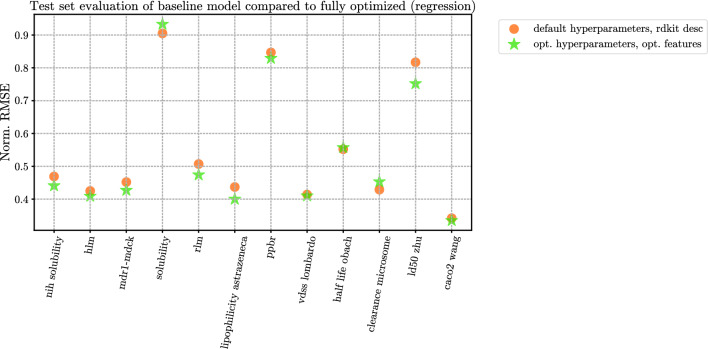
Comparison of baseline (CatBoost default hyperparameters + rdkit_desc features) versus fully optimized (CatBoost optimized hyperparameters + optimized features) on the test set of each dataset. Exact values, including all four model configurations, can be seen in Table 9. Pearson R values can be seen in Fig. 9

**Fig. 5:**
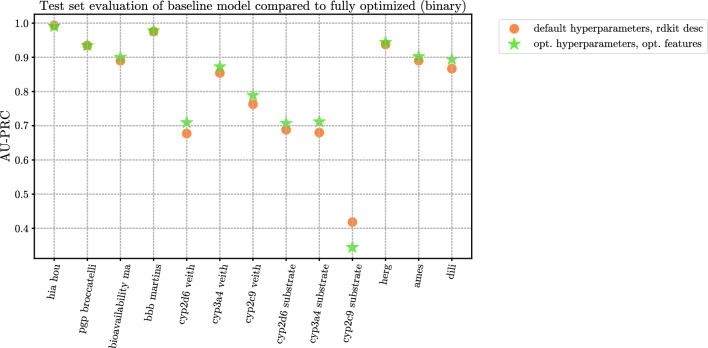
Comparison of baseline (CatBoost default hyperparameters + rdkit_desc features) versus fully optimized (CatBoost optimized hyperparameters + optimized features) on the test set of each dataset. Exact values, including all four model configurations, can be seen in Table 9. ROC-AUC values can be seen in Fig. 10

Finally, the four model configurations are evaluated on the test set, after training the models on the entire available training/validation data. The results are shown in Table 9. To visualize the impact of the entire optimization process, we visualize the test set NRMSE and AUPRC performances in Figs. 4 and 5 specifically for the baseline and fully optimized (optimized features + hyperparameter optimization) models. The same figures with Pearson R, ROCAUC values are available in Figs. 9 and 10, respectively.

For 19 out of 25 datasets, the optimizations have led to an improvement over the baseline model. This number jumps up to 21 out of 25 if either the non-optimized or the optimized catboost model with optimized features is considered, as opposed to the models using standard rdkit_desc or rdkit_desc + ecfp4 feature combinations. For 12 datasets, hyperparameter optimization leads to a decrease in test set performance compared to the non-optimized model with optimized features.

Only for 11 datasets the optimizations were statistically significant according to the Nemenyi test using a 10-fold CV method. In all 11 cases, an improvement on the test set performance was observed as well.

**Table 9:** Test set evaluation of model configurations at four different degrees of optimization

Dataset	catboost default, rdkit_desc	catboost default, rdkit_desc + ecfp4	catboost default, optimized features	catboost optimized, optimized features	10-fold CV Nemenyi test^a,b^ $p<0.05$	Test set improvement^c^
**TDCommons - regression**	**NRMSE** ($\downarrow$)					
caco2_wang	0.342	0.336	**0.323**	0.335	✓	✓
lipophilicity	0.437	0.428	0.408	**0.400**	✓	✓
ppbr_az	0.847	0.859	0.830	**0.829**	✗	✓
ld50_zhu	0.817	0.811	0.781	**0.752**	✓	✓
vdss_lombardo	0.414	0.412	0.411	**0.410**	✗	✓
half_life_obach	**0.552**	0.561	0.562	0.557	✗	✗
clearance_microsome	**0.429**	0.437	0.437	0.453	✗	✗
_az						
**TDCommons - binary**	**AUPRC** ($\uparrow$)					
bioavailability_ma	0.890	0.882	**0.909**	0.900	✗	✓
hia_hou	**0.994**	0.993	**0.994**	0.991	✗	✗
pgp_broccatelli	0.935	0.937	**0.939**	0.935	✗	✗
bbb_martins	0.976	0.979	**0.981**	0.978	✗	✓
cyp2c9_veith	0.763	0.773	0.778	**0.789**	✓	✓
cyp2d6_veith	0.677	0.694	**0.712**	0.709	✓	✓
cyp3a4_veith	0.854	**0.877**	0.874	0.873	✓	✓
cyp2c9_substrate	0.418	0.416	**0.448**	0.345	✗	✗
_carbonmangels						
cyp2d6_substrate	0.688	0.683	**0.719**	0.707	✗	✓
_carbonmangels						
cyp3a4_substrate	0.680	0.696	0.709	**0.712**	✗	✓
_carbonmangels						
herg	0.938	0.936	**0.957**	0.944	✗	✓
ames	0.890	0.896	0.896	**0.902**	✗	✓
dili	0.867	0.883	0.887	**0.894**	✗	✓
**NIH - regression**	**NRMSE** ($\downarrow$)					
solubility	0.469	0.462	0.457	**0.441**	✓	✓
**Biogen - regression**	**NRMSE** ($\downarrow$)					
rlm	0.507	0.489	0.485	**0.474**	✓	✓
solubility	0.905	0.911	**0.877**	0.933	✗	✗
hlm	0.425	0.422	0.424	**0.409**	✓	✓
mdr1-mdck	0.452	0.441	0.431	**0.427**	✓	✓

^a^In the 10-fold CV experiment, each configuration was trained 10 times for each dataset, allowing us to perform Nemenyi hypothesis test to compare the model configurations

^b^In the penultimate column, the outcome of the hypothesis ($p<0.05$ or not) is shown, specifically comparing the fully optimized (features and hyperparameters) model to the baseline model

^c^In the last column, we also denote whether the fully optimized configuration outperformed the baseline on the held-out test set

### Transferability

**Fig. 6:**
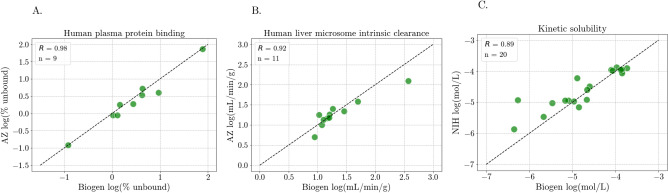
Correlation between the property values for overlapping compounds across the datasets for** A** hPPB,** B** HLM, and** C** solubility. Paired t-tests indicated p-values of 0.15, 0.35, and 0.12 respectively for hPPB, HLM, and solubility, thus the measurements from the different laboratories are not significantly different

The selected model configurations were subsequently evaluated in a more practical scenario, compared to the manually created test set splits. Multiple public datasets for the same property, but from different sources, were found for hPPB, HLM, and solubility. Respectively, 9, 11 and 20 overlapping compounds were found in the dataset pairs for hPPB, HLM and solubility. After adjusting the values to reflect similar units, Pearson correlations of 0.98, 0.92, and 0.89 were obtained for the respective properties of the overlapping compounds measured in different laboratories (Fig. 6). As pairwise t-tests indicated that the measurements are not significantly different (p-values > 0.1), we assessed the influence of using data from one laboratory on the predictive outcome of the properties measured in a different laboratory.

**Fig. 7:**
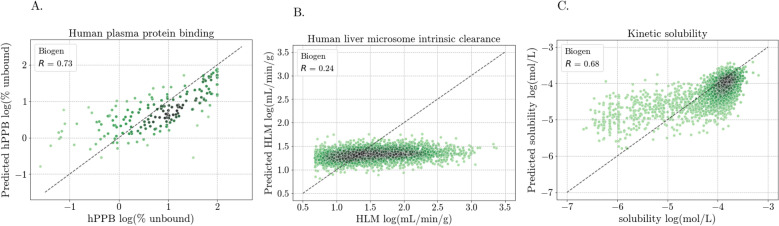
Correlation between the measured and predicted property values for models trained on data from one source and tested data from another across.** A** hPPB,** B** HLM, and** C** solubility

Firstly, the different levels of model optimization, the baseline catboost model compared to optimal feature combinations and hyperparameters, had minimal benefit when training on TDC data and testing on Biogen data for hPPB and HLM (Table 10). In the case of solubility, where models were trained with NIH data and tested on Biogen data, a decrease in RMSE was observed alongside the iteratively optimized features and hyperparameters. Looking at the correlation between the predicted and measured values indicates that the hPPB and solubility models tend to over-predict the lower values (Fig. 7), whereas the HLM model offered little advantage over just predicting the mean (1.3 log(mL/min/g).

**Table 10:** Performance of models at various levels of optimization when trained on data from one source (TDC or NIH) and evaluated on data from a different source (Biogen)

Features	Model	hPPB RMSE	HLM RMSE	Sol. RMSE
rdkit_	catboost	0.587	0.605	0.548
desc	default			
rdkit +	catboost	0.587	0.603	0.539
ecfp4	default			
optimized	catboost	0.596	0.600	0.526
features$^a$	default			
optimized	catboost	0.587	0.600	**0.523**
features$^a$	optimized			
	params			

$^a$ rdkit_desc, ecfp4, erg, and avalon fingerprints

Apart from differences in dataset sizes, we sought to see whether compound similarity between the training and test sets across the properties, and/or dataset distribution shifts would explain the difference in model performance between the properties. Tanimoto similarity (based on ecfp4) between test set (Biogen) compounds and that of the most and least similar compounds in the training sets (TDC/NIH), as well as the overall average compound similarity between test and training sets, are similar across the properties and does not explain model performance differences (Fig. 11). Distributions of the training and test set target values (the measured property values) are statistically different for all three properties (p-values are respectively 0.01, $\sim 10^{-39}$, and $\sim 10^{-56}$ for hPPB, HLM, and solubility). Albeit the distribution differences, the Biogen hPPB data falls within a similar range to that of the training TDC data, whereas Biogen HLM data goes beyond that of the TDC training data (Fig. 12). Though Biogen solubility data also ranges beyond the NIH training data, the range is overall well represented by the training data.

**Fig. 8:**
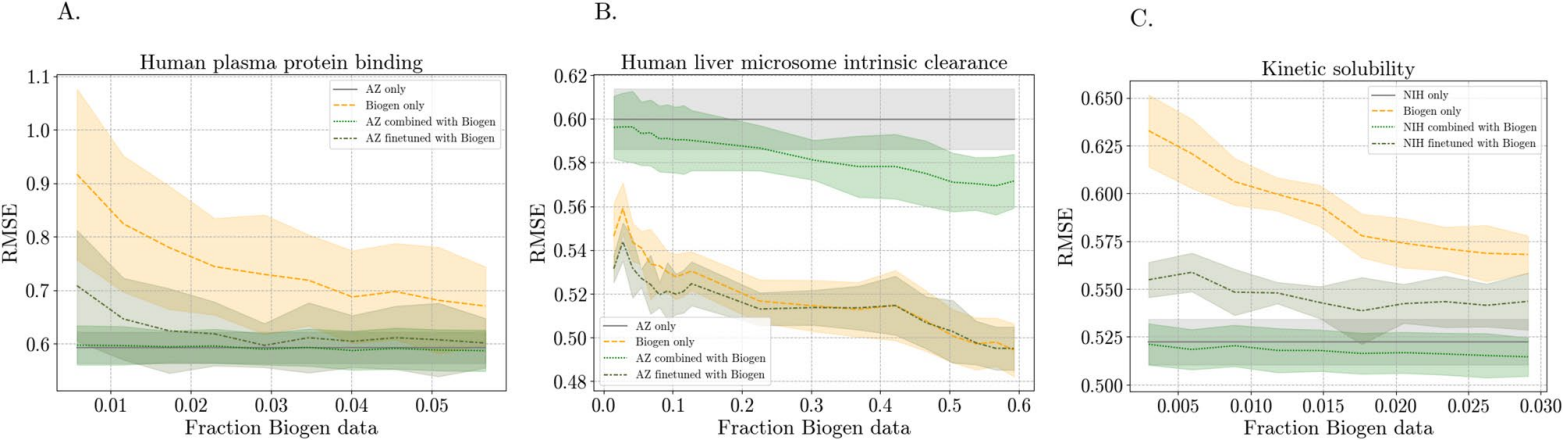
Performance of models trained with increasing amounts of Biogen data by itself, or combined with TDC or NIH data. The standard deviation across 5-folds are indicated by the shading around the means for experiments across** A** hPPB,** B** HLM, and** C** solubility. The grey baselines indicate models trained with TDC or NIH data only

**Fig. 9:**
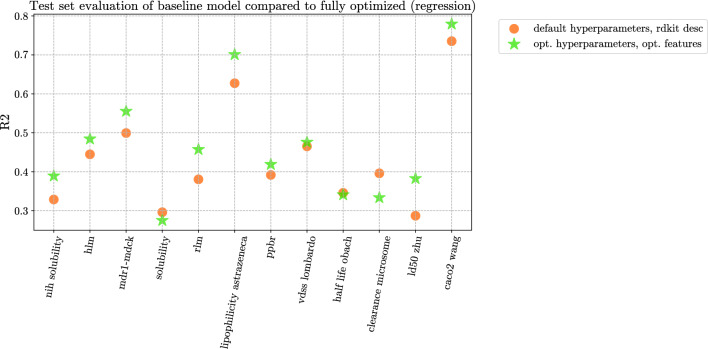
Comparison of baseline (CatBoost default hyperparameters + rdkit_desc features) versus fully optimized (CatBoost optimized hyperparameters + optimized features) on the test set of each dataset, evaluated via R2

**Fig. 10:**
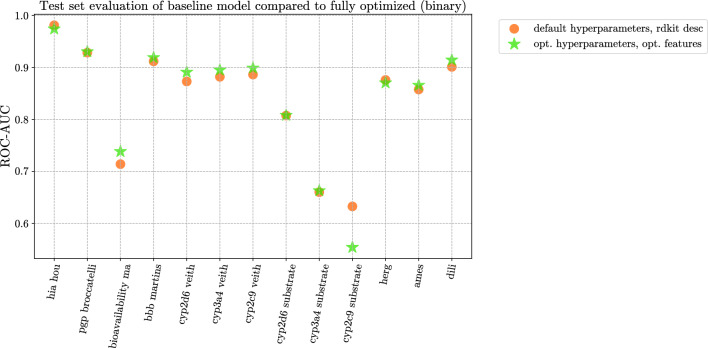
Comparison of baseline (CatBoost default hyperparameters + rdkit_desc features) versus fully optimized (CatBoost optimized hyperparameters + optimized features) on the test set of each dataset, evaluated via ROC-AUC

**Fig. 11:**
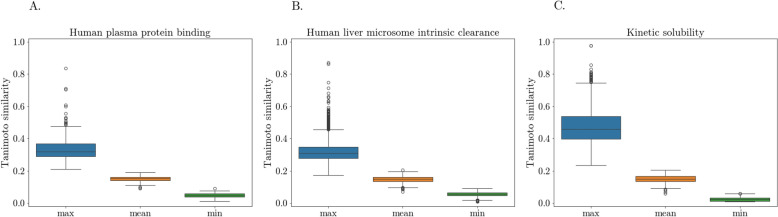
Distribution of Tanimoto similarity between compounds in the test set and those in the training set. For each compound in the test set, only the most similar (max) compound in the training set, the average similarity to all training set compounds (mean), and the least similar compound (min). Training data from one source and test data from another spans the properties.** A** hPPB,** B** HLM, and** C** solubility

We continued to use the optimized features and hyperparameters in further experiments to assess the influence of either including Biogen data in the training sets, finetuning, or adapting the training sets using Biogen data. Omitting, and then incrementally increasing the amount of Biogen data in the training sets of the models either does not affect, in the case of hPPB, or decrease RMSE as the Biogen proportion of training data increases compared to only using TDC or NIH data (Fig. 8, see also Fig. 14). The increasing effect is most pronounced in the case of HLM, where a proportionally higher amount of Biogen data is available and included (up to almost 60% of the training set), though it does not outperform using only Biogen HLM data. In all cases, when only using Biogen data for training, the increase in data decreases the RMSE. In the case of HLM, the additional TDC data shows some benefit when the model is trained using TDC data and finetuned with increments of Biogen data. However, as the Biogen data increases, the benefit of such finetuning diminishes. The combination of TDC or NIH data with Biogen data yields the lowest RMSEs for hPPB and kinetic solubility. Instance reweighting domain adaptation methods were also investigated as we expect covariate shifts between the datasets (Fig. 13). However, none of the adaptation methods outperformed the data combination or finetuning approaches.

## Discussion

The experiments in this study extensively explore ML models and features utilized in the ADMET space, focusing on statistical significance when comparing between them.

The initial model selection experiment via single-fold evaluation has shown that when using a single feature representation, catboost, svm and lightgbm all perform statistically similarly according to the Nemenyi post-hoc test. However, when two feature representations are used (rdkit_desc combined with other features), catboost comes out as the preferred model architecture, yielding significantly better performance with $p<0.05$ under the Nemenyi test.

random forest as well as mpnn model architectures performed poorly in our experiments. The poor performance of the mpnn is surprising considering it often is shown to yield good results in the literature [22, 42, 43]. This could be explained by the hands-off approach of our study, in which we did not supervise the training process of either model, simply making use of the training and prediction CLI scripts as instructed without any hyperparameter tuning. Deep learning models often require some supervision besides the early stopping criteria to ensure that the training has converged properly. Moreover, deep learning models typically require more data compared to decision tree based architectures. Therefore, we investigate the differences in model performance between the baseline mpnn and catboost models trained with rdkit_desc features, with the dataset size in mind. This data can be seen in Table 11. We observe that as the dataset size increases, the model performances become more and more comparable, with mpnn ultimately outperforming baseline catboost on the NIH solubility dataset containing $\sim$36k compounds.

The feature performance investigation has showed that using the rdkit_desc representation of compounds is a safe choice for molecular property prediction tasks in the ADMET domain, performing significantly better used as a single feature compared to any other single feature across all models. However, it is a much more reliable representation for regression tasks compared to binary classification, attaining average ranks of 1.91 and 4.31 respectively across pairs of dataset-model combinations for both task types.

Iterative feature addition (Tables 6 and 7) has resulted in combinations that yield significantly better performance for both regression as well as binary classification tasks across the datasets. In both cases, using only rdkit_desc features, or a common rdkit_desc + ecfp4 feature combination does not bring out the best possible model performance in the datasets. The improvement that comes from using the optimized set of features is more significant than the improvement of subsequent hyperparameter optimization, as seen both in the cross-validation analysis (Table 8) as well as the test set evaluation (Table 9). Moreover, adding extra features results in decreased performance, suggesting that the added noise outweighs the additional representational power.

The optimal sets of features for regression^3^ and binary classification^4^ tasks contain only standard cheminformatics representations, showing that deep-learning based features (both LLM and graph-based) do not perform well in the ADMET domain. Notably, as a standalone feature, megamolbart has achieved an average ranking of 4.46 in the binary classification datasets, compared to the 4.31 of rdkit_desc, which is the only time in the study where a deep-learning based feature representation has come close to the standard cheminformatics ones. A possible interpretation for why deep-learning features do not perform well in this domain is the high-noise, low-data issue: the vast information encoded in the high-dimensional compound embeddings of deep-learning models might be too hard to uncover when guided only by the highly noisy and sparse data.

To better understand the performance gap between rdkit_desc and deep learning features, we identified the most influential descriptors using SHAP [44] and selected them for further analysis. Our hypothesis is that deep learning features do not effectively encode the most impactful rdkit_desc features. To test this, we performed separate regressions, using the catboost model, with each deep learning embedding to predict each of the top five rdkit_desc features for each dataset (some were shared across datasets). For example, regressing to the value of the *Number-of-Aromatic-Heterocycles* rdkit_desc feature from each of the deep-learning embeddings. As shown in Fig. 15, the Deep Learning embeddings with CatBoost generally struggle with this task. The exception is Grover-the only graph-based embedding-which likely performs better in capturing the physicochemical properties of molecules, in contrast to the Large Language Model (LLM) based approaches in the other Deep Learning embeddings. However, the ability of Grover embeddings to be used to predict rdkit_desc features does not necessarily imply their effectiveness in modeling ADMET properties. For further feature analyses, see Figs. 16, 17, 18, 19, 20, 21.

Overall, the model optimization has been impactful in increasing the test set performance. Non-optimized models with either rdkit_desc or rdkit_desc+ecfp4 features only performed best on 4 out of 25 test sets, whereas one of the optimized model configurations performed the best on the remaining 21. Interestingly, while the hyperparameter optimization was shown to be statistically significant in the cross-validation experiment, it had mixed results in increasing the performance on the test set, where for 12 datasets it has led to a decrease in performance. However, only 3 of the 12 datasets were regression datasets, suggesting that the optimization is less robust in the binary classification setting.

In every set of comparisons, the Friedman $\chi ^2$ test was easily passed with $p<0.01$; however, the post-hoc Nemenyi test did not always identify significant differences under $p<0.05$. In particular, passing the 10-fold cross-validation hypothesis test is harder than improving the test set performance; often even when a substantial improvement in the test set is observed, the hypothesis test did not pass with $p<0.05$. However, for the 11 datasets where the hypothesis test was passed, a test set improvement was observed too. These results suggest that hypothesis testing can be utilized as a more robust alternative to single hold-out set evaluation, allowing to identify model improvements that are much more likely to generalize. A case for when such a robustness can be useful is seen in the results of the transferability experiment.

Out of the three datasets used in the transferability experiment, model optimization yielded a noticeable improvement only in the solubility dataset, as seen in Table 10. This aligns with our test set observations (Table 9): for clearance_microsome_az, neither the hypothesis test was passed nor a test set improvement was observed, whereas the opposite was true for the NIH solubility dataset. The remaining dataset presents an argument for why the robustness of hypothesis might be preferred to a test set evaluation: for the ppbr_az dataset, we observe an improvement in the test set. We might therefore incorrectly expect an improvement in the transferability experiment. However, the hypothesis test was rejected; hence if we were to be guided by the hypothesis test, the lack of improvement is no surprise.

**Fig. 12:**
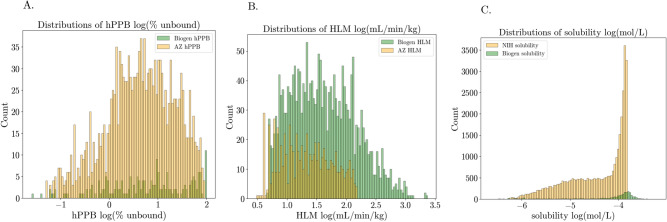
Distributions of measured values (dependent variables) between compounds in the test set (Biogen) and those in the training set (AZ or NIH). Training data from one source and test data from another spans the properties.** A** hPPB (p-value=0.01),** B** HLM (p-value$\sim 10^{-39}$), and C) solubility (p-value$\sim 10^{-56}$)

**Fig. 13:**
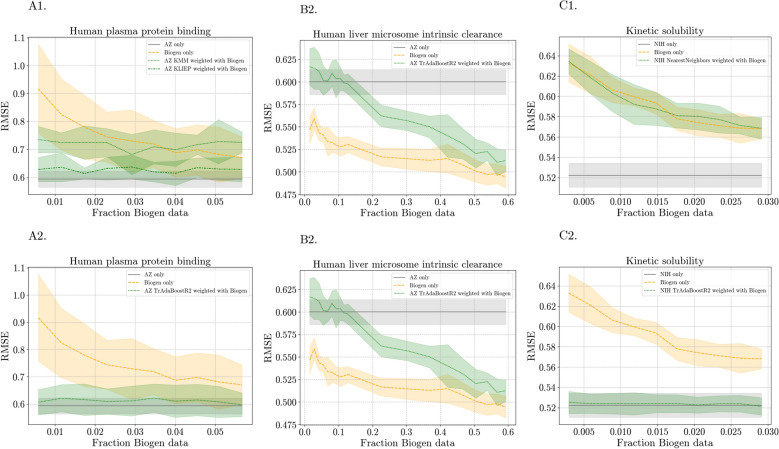
Performance of models adapted with increasing amounts of Biogen compared to Biogen data by itself. The first panels display results from unsupervised adaptation methods relevant to the dataset size, whereas the second panels display supervised adaptation methods across the properties.** A** hPPB,** B** HLM, and** C** solubility. The grey baselines indicate models trained with TDC or NIH data only without any adaptation

**Fig. 14:**
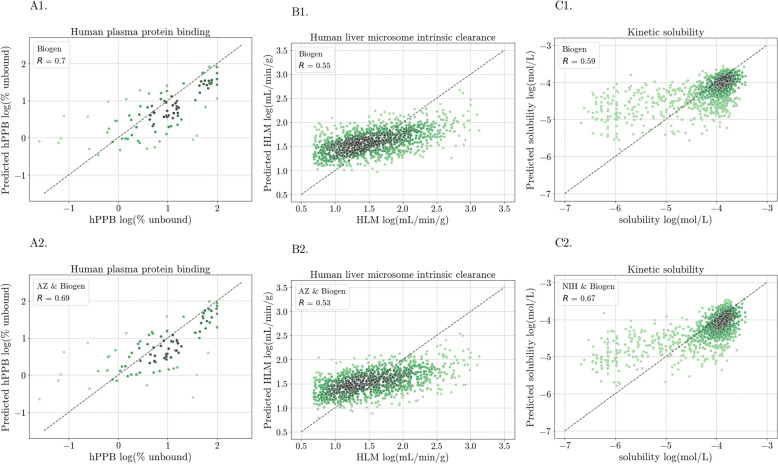
Correlation between predicted and measured values from models trained with 50% Biogen data (1st panels), and the 50% Biogen data combined with the TDC or NIH data (2nd panels) across** A** hPPB,** B** HLM, and** C** solubility

**Fig. 15:**
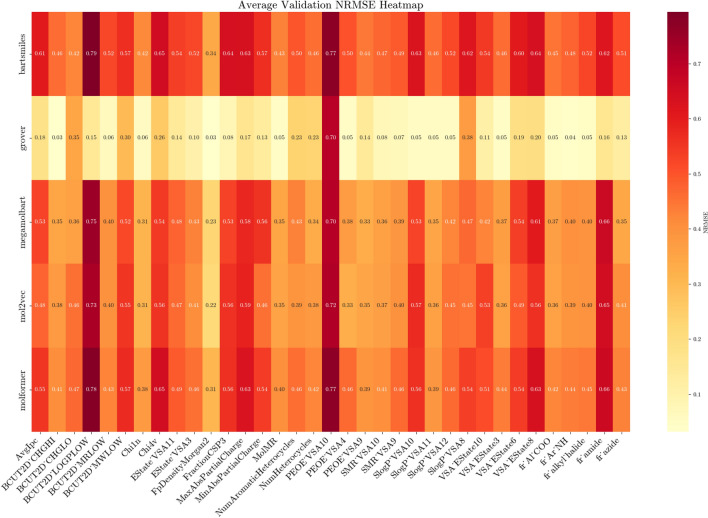
Heatmap of deep learning embeddings and their ability to predict the most important rdkit_desc features

**Fig. 16:**
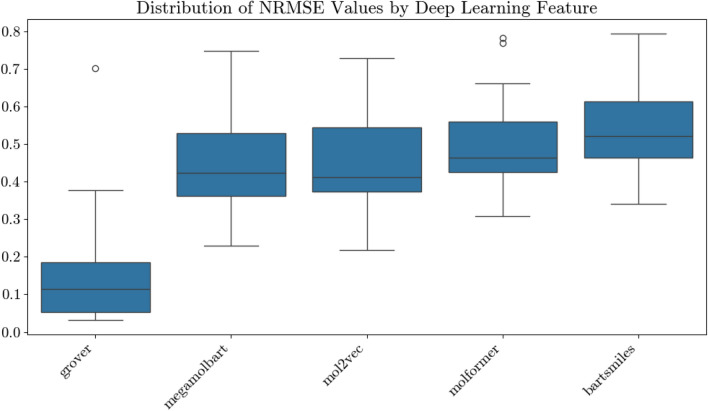
Boxplot of deep learning embeddings and their ability to predict the most important rdkit_desc features, aggregated across datasets and feature types

**Fig. 17:**
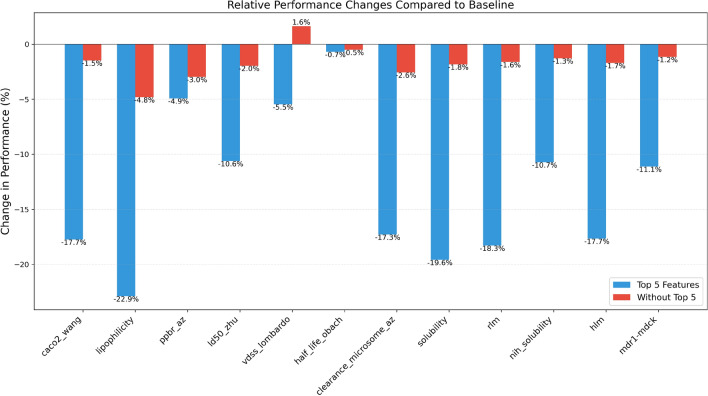
Changes in model performance when using only top 5 (blue) and all expect the top 5 (red) most important features per dataset

**Fig. 18:**
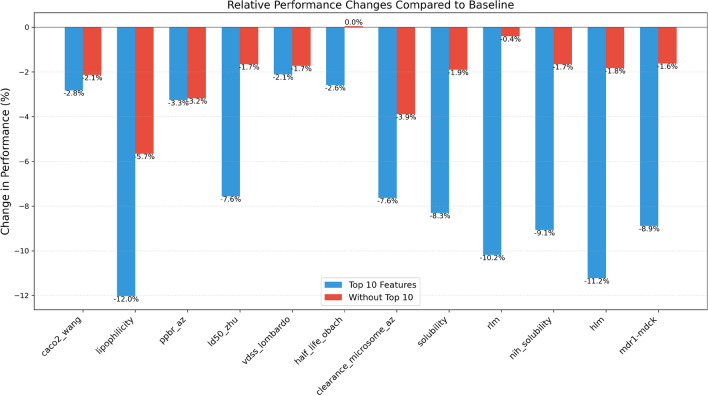
Changes in model performance when using only top 10 (blue) and all expect the top 10 (red) most important features per dataset

**Fig. 19:**
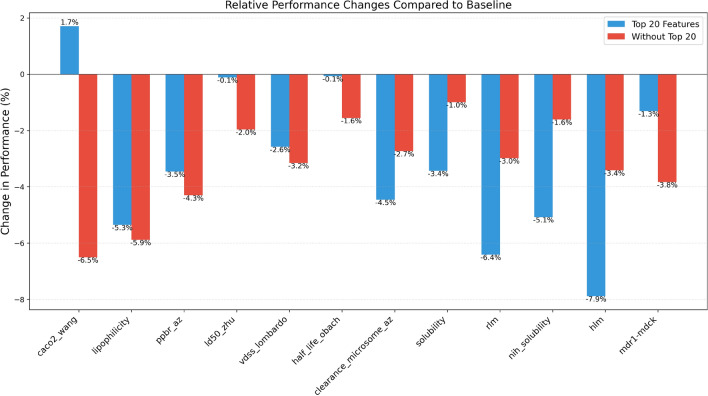
Changes in model performance when using only top 20 (blue) and all expect the top 20 (red) most important features per dataset

**Fig. 20:**
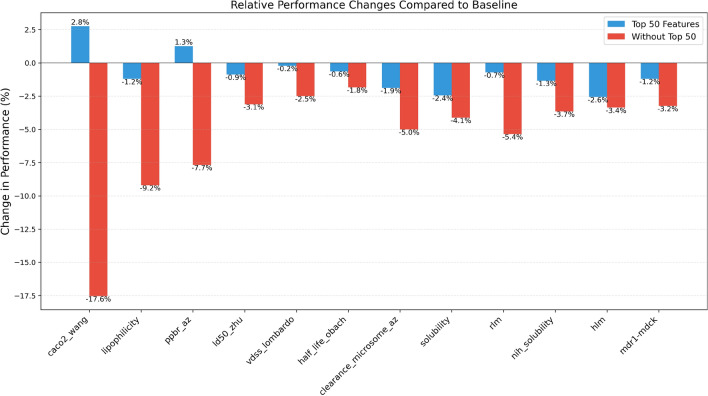
Changes in model performance when using only top 50 (blue) and all expect the top 50 (red) most important features per dataset

**Fig. 21:**
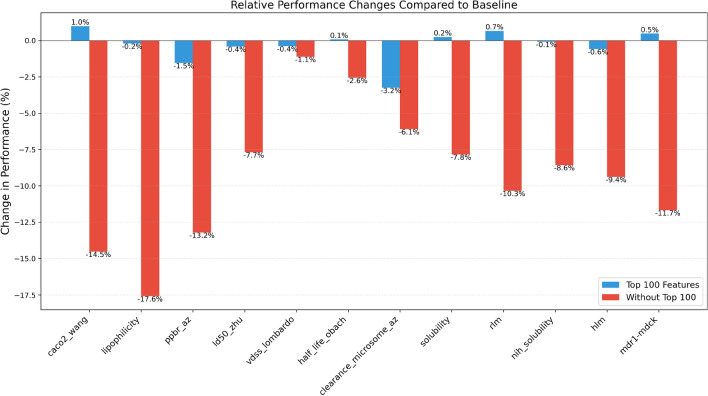
Changes in model performance when using only top 100 (blue) and all expect the top 100 (red) most important features per dataset

Provided that the assay conditions and endpoints are similar, using data from a different laboratory in predictive models could be beneficial when no data from the laboratory in question, represented by Biogen data in this study, is available (Fig. 7). The performance of the models seem to be property dependent, with similarity to compounds in the training set not providing any indication of whether the model would perform well. Even though all three properties have statistically significant differences in the distributions of their measured property values, considering these differences in more detail can illuminate the differing transferability outcomes. First of all, the Biogen property range for the HLM data has a significant proportion of the values lying outside of the TDC data range, compounded by the bigger Biogen HLM dataset size compared to TDC HLM data. While this is also the case for solubility, the Biogen solubility values falling outside the NIH range are close (within 0.3 in log scale) to the NIH limit of − 3.8, which is very well represented (Fig. 12). In contrast, the Biogen data range for hPPB is much closer than the other two datasets, reflected by the t-test p-value of 0.01, which is considerably larger than the p-values for the other two datasets (orders of $10^{-39}$ and $10^{-56}$).

Though the small sample of overlapping HLM compounds have similar measurements between the laboratories (Fig. 6), they are almost all within the TDC training data IQR (1.0−1.7 log(mL/min/kg) Fig. 12), which is the range predicted when training solely on TDC HLM data (Fig. 7). These overlapping compounds might thus not be sufficient to indicate HLM measurement differences across the full property range, nor indicate out of range compounds. We know that HLM experiments have numerous assay variables that can differ between laboratories and influence measured target values [45], hence without a wider set of overlapping compounds we cannot be certain of experimental data alignment.

The combination of TDC and Biogen data proved to be the best data integration approach for hPPB and solubility. It is clear from hPPB that the benefit of additional TDC data decreases as more Biogen data is included in model training (Fig. 8). In part, it appears to depend on the amount of additional data compared to Biogen data. For hPPB, the use of the additional data starts to become insignificant, as indicated by overlapping standard deviation, when around 5% or more Biogen data is available (Fig. 8). In the case of solubility, a similar trend is apparent. However, as much more NIH than Biogen data is available for solubility, the point at which the additional NIH data becomes insignificant was not reached and can thus not be concluded.

The best use of the additional TDC HLM data was by means of using it to construct a base model that could further be finetuned using Biogen data. Finetuning might thus be the better approach when there is more uncertainty about assay similarity. As with the other properties, the benefit of the additional data diminished as the Biogen data increased, again highlighting the advantage of internal data.

## Conclusions

This study provides a detailed analysis of compound representations and machine learning techniques in ADMET tasks. The systematic approach taken across baseline model and feature selection, and the incorporation of statistical methods in comparisons, advances the accuracy and reliability of ML applications in the ADMET molecular property space. Furthermore, the systematic approach can reveal the degree of benefit afforded by the integrated use of data from different sources.

## Data Availability

The data used in this study is made available via the publicly available GitHub repository: https://github.com/Ro5-ai/bio2d_public. The downloadable data file contains the datasets listed in Table 1, containing the collected publicly available datasets that were cleaned as described in tables 18, 19, 20, 21, 22, 23, 24, 25, 26, 27, 28, 29, 30, 31, 32, 33, 34, 35, 36, 37, 38, 39, 40, 41, 42. The data file also contains the pre-computed featurizations of the compounds, using all the featurization methods used in this study. The associated codebase contains model training code, allowing one to train any model used in this study with any set of features on any dataset. Moreover, it contains the transferability experiment framework, allowing the user to train a source-data model with iteratively more domain data using various methods explored in this paper.

## Appendix

### Test set evaluation over different metrics

In the main body of paper, only NRMSE and AUPRC values are used for the datasets. Here we include the test set evaluations on the regression and binary classification tasks using other two popular metrics, $R^2$ and ROC-AUC, respectively.

### MPNN versus CatBoost comparison

See Table 11

A common notion is that deep learning models require more data to be trained compared to decision tree based architectures. Therefore, we investigate the differences in model performance between the baseline mpnn and catboost models trained with rdkit_desc features, with the dataset size in mind. This data can be seen in Table 11. We observe that as the dataset size increases, the model performances become more and more comparable.

**Table 11:** mpnn and catboost performance comparison on the regression datasets, based on the dataset sizes

Dataset name	Dataset size	mpnn $R^2$	catboost $R^2$
half_life_obach	662	0.16	**0.25**
caco2_wang	753	0.52	**0.61**
clearance_microsome_az	1102	0.26	**0.32**
vdss_lombardo	1105	0.35	**0.45**
ppbr_az	1614	0.37	**0.44**
solubility	2173	**0.28**	**0.28**
mdr1-mdck	2642	0.43	**0.50**
rlm	3054	**0.47**	**0.49**
hlm	3087	**0.39**	**0.40**
ld50_zhu	7323	0.14	**0.25**
nih_solubility	36238	**0.32**	0.27

The results are based on the single-fold hold-out set experiment (validation, not test)

### Additional transferability information

The figures below shows the Tanimoto similarity distributions (Fig. 11) of test set compounds from one source to that for training set compounds from a different source as discusses in the transferability experiment. Further, we include the predicted vs. measured correlations (Fig. 14) for training sets consisting of 50% of the Biogen data, and also the combination of the 50% Biogen data and the TDC (hPPB and HLM) or NIH (solubility) data respectively.

### Feature prodding

This experiment evaluates the extent to which classical molecular descriptors (rdkit_desc) can be predicted from deep learning-derived molecular representations. The experimental procedure can be split into two parts:

#### Feature attribution

For each dataset, a catboost regressor is trained using rdkit_desc features to predict the target. Model performance is assessed using NRMSE on training and validation splits. SHAP [44] (SHapley Additive exPlanations) values are then computed to rank features by importance. The top five descriptors are selected based on their SHAP values.

#### Feature prodding

For each of the five main descriptors, a series of regression models are trained to predict the value of the descriptor from different sets of deep learning embeddings. Each model uses a deep learning feature representation as input and the selected descriptor as the regression target. A separate catboost regressor is trained for each combination of descriptor and feature set.

This approach quantifies how well various deep learning embeddings encode the most chemically relevant information for each dataset. Model performance is evaluated using NRMSE on the validation splits. Results are aggregated for comparison across datasets and feature types and can be seen in Figs. 15, 16, 17, 18 ,19, 20 and 21.

### Default hyperparameters

T﻿he tables in this section show the default hyperparameters used by each model in the Model Selection stage of the experimentation. See Tables 12, 13, 14, 15, 16, 17, 18.

**Table 12:** Default CatBoost hyperparameters (Python package)

Parameter	Default value
iterations	1000
learning_rate	0.03
depth	6
l2_leaf_reg	3.0
bootstrap_type	Bayesian
loss_function	RMSE (Regressor) | Logloss (Classifier)
random_seed	None (0)
bagging_temperature	1

**Table 13:** Default LightGBM core learning hyperparameters

Parameter	Default value
num_iterations	100
learning_rate	0.1
boosting	gbdt
num_leaves	31
max_depth	$-1$ (unlimited)
min_data_in_leaf	20
feature_fraction	1.0
bagging_fraction	1.0
bagging_freq	0
lambda_l1	0.0
lambda_l2	0.0
objective	regression

**Table 14:** Default D-MPNN architecture hyperparameters (Chemprop v1.6.1)

Parameter	Default value
hidden_size	300
depth (message passes)	3
ffn_num_layers	2
ffn_hidden_size	inherits hidden_size (if None)
dropout	0.0
activation	ReLU
bias	False
atom_messages	False
undirected	False
aggregation	mean
aggregation_norm	100 *(only if aggregation = norm)*
mpn_shared	False
ensemble_size	1

**Table 15:** Default training hyperparameters (Chemprop v1.6.1)

Parameter	Default value
batch_size	50
epochs	30
warmup_epochs	2.0
init_lr	$1\times 10^{-4}$
max_lr	$1\times 10^{-3}$
final_lr	$1\times 10^{-4}$
grad_clip	None
num_workers	8
seed / pytorch_seed	0
class_balance	False

**Table 16:** Default hyperparameters of sklearn.ensemble.RandomForestClassifier

Parameter	Default value
n_estimators	100
criterion	gini
max_depth	None
min_samples_split	2
min_samples_leaf	1
max_features	sqrt
bootstrap	True
oob_score	False
n_jobs	None
class_weight	None
random_state	None

**Table 17:** Default hyperparameters of sklearn.svm.SVC (v1.6)

Parameter	Default value
C	1.0
kernel	rbf
degree	3
gamma	scale
coef0	0.0
shrinking	True
probability	False
tol	0.001
cache_size	200
class_weight	None
max_iter	$-1$ (no limit)
decision_function_shape	ovr
break_ties	False
random_state	None

### Data cleaning

The tables below show all the dataset-specific information behind the data cleaning that was carried out. See Tables 19, 20, 21, 22, 23, 24, 25, 26, 27, 28, 29, 30, 31, 32, 33, 34, 35, 36, 37, 38, 39, 40, 41, 42.

**Table 18:** Data cleaning breakdown for rlm

Metric	Total	Unchanged	Transformed	Removed
SMILES count	3054	2991	63	0

Transformation description	Count			
02 2-hydroxy pyridine -> 2-pyridone	30			
canonicalized	21			
03 4-hydroxy pyridine -> 4-pyridone (within-ring)	6			
25 Charge-seperate sulphoxides	5			
04 4-pyrimidone -> 2-pyrimidone (any)	1

**Table 19:** Data cleaning breakdown for solubility

Metric	Total	Unchanged	Transformed	Removed
SMILES count	2173	2140	33	0

Transformation description	Count			
02 2-hydroxy pyridine -> 2-pyridone	18			
canonicalized	7			
25 Charge-seperate sulphoxides	4			
03 4-hydroxy pyridine -> 4-pyridone (within-ring)	4

**Table 20:** Data cleaning breakdown for mdr1-mdck

Metric	Total	Unchanged	Transformed	Removed
SMILES count	2642	2587	55	0

Transformation description	Count			
02 2-hydroxy pyridine -> 2-pyridone	26			
canonicalized	17			
03 4-hydroxy pyridine -> 4-pyridone (within-ring)	7			
25 Charge-seperate sulphoxides	4			
04 4-pyrimidone -> 2-pyrimidone (any)	1

**Table 21:** Data cleaning breakdown for hlm

Metric	Total	Unchanged	Transformed	Removed
SMILES count	3087	3023	64	0

Transformation description	Count			
02 2-hydroxy pyridine -> 2-pyridone	30			
canonicalized	21			
03 4-hydroxy pyridine -> 4-pyridone (within-ring)	7			
25 Charge-separate sulphoxides	5			
04 4-pyrimidone -> 2-pyrimidone (any)	1

**Table 22:** Data cleaning breakdown for caco2_wang

Metric	Total	Unchanged	Transformed	Removed
SMILES count	910	587	45	278

Removal reason	Count			
inconsistent_duplicate	256			
duplicate	21			
multi_component	1			

Transformation description	Count			
canonicalized	30			
02 2-hydroxy pyridine -> 2-pyridone	4			
25 Charge-seperate sulphoxides	4			
13 Enol -> Ketone 1	3			
04 4-pyrimidone -> 2-pyrimidone (any)	3			
03 4-hydroxy pyridine -> 4-pyridone (within-ring)	1

**Table 23:** Data cleaning breakdown for ld50_zhu

Metric	Total	Unchanged	Transformed	Removed
SMILES count	7385	7221	87	77

Removal reason	Count			
inconsistent_duplicate	66			
duplicate	8			
no_non_salt_or_inorganic	3			

Transformation description	Count			
25 Charge-seperate sulphoxides	48			
13 Enol -> Ketone 1	22			
04 4-pyrimidone -> 2-pyrimidone (any)	8			
canonicalized	3			
03 4-hydroxy pyridine -> 4-pyridone (within-ring)	3			
10 Fix heterocyclic tautomer 2	2			
02 2-hydroxy pyridine -> 2-pyridone	1

**Table 24:** Data cleaning breakdown for dili

Metric	Total	Unchanged	Transformed	Removed
SMILES count	475	442	24	9

Removal reason	Count			
multi_component	6			
multi_organic_salt	2			
no_non_salt_or_inorganic	1			

Transformation description	Count			
canonicalized	11			
13 Enol -> Ketone 1	7			
25 Charge-seperate sulphoxides	4			
04 4-pyrimidone -> 2-pyrimidone (any)	2

**Table 25:** Data cleaning breakdown for ames

Metric	Total	Unchanged	Transformed	Removed
SMILES count	7278	7069	151	58

Removal reason	Count			
inconsistent_duplicate	38			
duplicate	16			
no_non_salt_or_inorganic	4			

Transformation description	Count			
02 2-hydroxy pyridine -> 2-pyridone	42			
canonicalized	35			
13 Enol -> Ketone 1	24			
10 Fix heterocyclic tautomer 2	14			
25 Charge-seperate sulphoxides	12			
11 Fix heterocyclic tautomer 3	11			
03 4-hydroxy pyridine -> 4-pyridone (within-ring)	9			
11 Fix heterocyclic tautomer 3 and				
02 2-hydroxy pyridine -> 2-pyridone	1			
07 hydropyridin-4-imine -> 4-amino-pyridine	1			
21 Fix 1,3 conjugated cation (non-aromatic)	1			
14 Enol -> Ketone 2	1

**Table 26:** Data cleaning breakdown for herg

Metric	Total	Unchanged	Transformed	Removed
SMILES count	655	333	270	52

Removal reason	Count			
duplicate	29			
inconsistent_duplicate	16			
multi_component	6			
no_non_salt_or_inorganic	1			

Transformation description	Count			
canonicalized	260			
25 Charge-seperate sulphoxides	5			
04 4-pyrimidone -> 2-pyrimidone (any)	2			
13 Enol -> Ketone 1	2			
02 2-hydroxy pyridine -> 2-pyridone	1

**Table 27:** Data cleaning breakdown for nih_solubility

Metric	Total	Unchanged	Transformed	Removed
Dataset count	36240	35775	463	2

Transformation description	Count			
04 4-pyrimidone -> 2-pyrimidone (any)	317			
14 Enol -> Ketone 2	73			
13 Enol -> Ketone 1	46			
25 Charge-separate sulphoxides	16			
canonicalized	7			
11 Fix heterocyclic tautomer 3	3			
04 4-pyrimidone -> 2-pyrimidone (any);14 Enol -> Ketone 2	1

**Table 28:** Data cleaning breakdown for clearance_microsome_az

Metric	Total	Unchanged	Transformed	Removed
SMILES count	1102	1065	37	0

Transformation description	Count			
02 2-hydroxy pyridine -> 2-pyridone	27			
canonicalized	7			
03 4-hydroxy pyridine -> 4-pyridone (within-ring)	3

**Table 29:** Data cleaning breakdown for half_life_obach

Metric	Total	Unchanged	Transformed	Removed
SMILES count	667	595	68	4

Removal reason	Count			
inconsistent_duplicate	2			
multi_component	1			
duplicate	1			

Transformation description	Count			
canonicalized	48			
13 Enol -> Ketone 1	12			
02 2-hydroxy pyridine -> 2-pyridone	5			
04 4-pyrimidone -> 2-pyrimidone (any)	2			
06 hydropyridin-2-imine -> 2-amino-pyridine (N-subst.)	1

**Table 30:** Data cleaning breakdown for cyp2c9_substrate_carbonmangels

Metric	Total	Unchanged	Transformed	Removed
SMILES count	669	613	53	3
Removal reason	Count			
duplicate	3			
Transformation description	Count			
canonicalized	40			
13 Enol -> Ketone 1	8			
04 4-pyrimidone -> 2-pyrimidone (any)	2			
25 Charge-separate sulphoxides	1			
01 hydroxy imine -> carboxamide	1			
02 2-hydroxy pyridine -> 2-pyridone	1

**Table 31:** Data cleaning breakdown for cyp3a4_substrate_carbonmangels

Metric	Total	Unchanged	Transformed	Removed
SMILES count	670	614	53	3
Removal reason	Count			
duplicate	3			
Transformation description	Count			
canonicalized	40			
13 Enol -> Ketone 1	8			
04 4-pyrimidone -> 2-pyrimidone (any)	2			
25 Charge-separate sulphoxides	1			
02 2-hydroxy pyridine -> 2-pyridone	1			
01 hydroxy imine -> carboxamide	1

**Table 32:** Data cleaning breakdown for cyp2d6_substrate_carbonmangels

Metric	Total	Unchanged	Transformed	Removed
SMILES count	667	612	51	4

Removal reason	Count			
duplicate	2			
inconsistent_duplicate	2			

Transformation description	Count			
canonicalized	38			
13 Enol -> Ketone 1	8			
04 4-pyrimidone -> 2-pyrimidone (any)	2			
25 Charge-seperate sulphoxides	1			
02 2-hydroxy pyridine -> 2-pyridone	1			
01 hydroxy imine -> carboxamide	1

**Table 33:** Data cleaning breakdown for cyp2c9_veith

Metric	Total	Unchanged	Transformed	Removed
SMILES count	12092	10958	969	165

Removal reason	Count			
multi_component	76			
no_non_salt_or_inorganic	43			
duplicate	23			
inconsistent_duplicate	19			
manual_inspection	2			
sanity_check	1			
multi_organic_salt	1			

Transformation description	Count			
canonicalized	833			
13 Enol -> Ketone 1	97			
25 Charge-seperate sulphoxides	28			
14 Enol -> Ketone 2	5			
07 hydropyridin-4-imine -> 4-amino-pyridine	2			
02 2-hydroxy pyridine -> 2-pyridone	2			
13 Enol -> Ketone 1;14 Enol -> Ketone 2	1			
01 hydroxy imine -> carboxamide	1

**Table 34:** Data cleaning breakdown for cyp3a4_veith

Metric	Total	Unchanged	Transformed	Removed
SMILES count	12328	11246	924	158

Removal reason	Count			
multi_component	78			
no_non_salt_or_inorganic	42			
inconsistent_duplicate	22			
duplicate	14			
manual_inspection	1			
multi_organic_salt	1			

Transformation description	Count			
canonicalized	785			
13 Enol -> Ketone 1	96			
25 Charge-seperate sulphoxides	31			
14 Enol -> Ketone 2	6			
07 hydropyridin-4-imine -> 4-amino-pyridine	3			
01 hydroxy imine -> carboxamide	1			
02 2-hydroxy pyridine -> 2-pyridone	1			
13 Enol -> Ketone 1;14 Enol -> Ketone 2	1

**Table 35:** Data cleaning breakdown for cyp2d6_veith

Metric	Total	Unchanged	Transformed	Removed
SMILES count	13130	11962	998	170

Removal reason	Count			
multi_component	80			
no_non_salt_or_inorganic	40			
inconsistent_duplicate	30			
duplicate	17			
manual_inspection	1			
sanity_check	1			
multi_organic_salt	1			

Transformation description	Count			
canonicalized	830			
13 Enol -> Ketone 1	118			
25 Charge-seperate sulphoxides	39			
14 Enol -> Ketone 2	6			
07 hydropyridin-4-imine -> 4-amino-pyridine	3			
13 Enol -> Ketone 1;14 Enol -> Ketone 2	1			
01 hydroxy imine -> carboxamide	1

**Table 36:** Data cleaning breakdown for vdss_lombardo

Metric	Total	Unchanged	Transformed	Removed
SMILES count	1130	394	711	25

Removal reason	Count			
duplicate	15			
inconsistent_duplicate	10			

Transformation description	Count			
canonicalized	673			
13 Enol -> Ketone 1	16			
25 Charge-seperate sulphoxides	10			
04 4-pyrimidone -> 2-pyrimidone (any)	7			
06 hydropyridin-2-imine -> 2-amino-pyridine (N-subst.)	2			
03 4-hydroxy pyridine -> 4-pyridone (within-ring)	2			
10 Fix heterocyclic tautomer 2	1

**Table 37:** Data cleaning breakdown for ppbr_az

Metric	Total	Unchanged	Transformed	Removed
SMILES count	1614	1572	42	0

Transformation description	Count			
02 2-hydroxy pyridine -> 2-pyridone	19			
canonicalized	11			
03 4-hydroxy pyridine -> 4-pyridone (within-ring)	10			
01 hydroxy imine -> carboxamide	2

**Table 38:** Data cleaning breakdown for bbb_martins

Metric	Total	Unchanged	Transformed	Removed
SMILES count	2030	1743	202	85

Removal reason	Count			
duplicate	44			
inconsistent_duplicate	36			
multi_component	5			

Transformation description	Count			
canonicalized	159			
13 Enol -> Ketone 1	28			
04 4-pyrimidone -> 2-pyrimidone (any)	9			
25 Charge-seperate sulphoxides	6

**Table 39:** Data cleaning breakdown for bioavailability_ma

Metric	Total	Unchanged	Transformed	Removed
SMILES count	640	580	58	2

Removal reason	Count			
no_non_salt_or_inorganic	1			
multi_component	1			

Transformation description	Count			
canonicalized	36			
13 Enol -> Ketone 1	10			
25 Charge-seperate sulphoxides	6			
04 4-pyrimidone -> 2-pyrimidone (any)	3			
02 2-hydroxy pyridine -> 2-pyridone	2			
06 hydropyridin-2-imine -> 2-amino-pyridine (N-subst.)	1

**Table 40:** Data cleaning breakdown for pgp_broccatelli

Metric	Total	Unchanged	Transformed	Removed
SMILES count	1218	1157	55	6

Removal reason	Count			
duplicate	6			

Transformation description	Count			
canonicalized	44			
13 Enol -> Ketone 1	5			
04 4-pyrimidone -> 2-pyrimidone (any)	3			
25 Charge-separate sulphoxides	2			
01 hydroxy imine -> carboxamide	1

**Table 41:** Data cleaning breakdown for hia_hou

Metric	Total	Unchanged	Transformed	Removed
SMILES count	578	529	49	0

Transformation description	Count			
canonicalized	29			
25 Charge-seperate sulphoxides	8			
13 Enol -> Ketone 1	7			
01 hydroxy imine -> carboxamide	4			
02 2-hydroxy pyridine -> 2-pyridone	1

**Table 42:** Data cleaning breakdown for lipophilicity

Metric	Total	Unchanged	Transformed	Removed
SMILES count	4200	4094	105	1

Removal reason	Count			
no_non_salt_or_inorganic	1			

Transformation description	Count			
02 2-hydroxy pyridine -> 2-pyridone	63			
03 4-hydroxy pyridine -> 4-pyridone (within-ring)	37			
01 hydroxy imine -> carboxamide	2			
25 Charge-seperate sulphoxides	1			
canonicalized	1			
04 4-pyrimidone -> 2-pyrimidone (any)	1			

## Footnotes

1 Note this technically makes our results incomparable to any of the public leaderboard models, even though the number of removed compounds in the test sets is minor.

2 mordred features were not combined with rdkit_desc because the mordred descriptors already contain rdkit_desc, along with a number of other features.

3 regression: rdkit_desc + erg + ecfp4 + avalon

4 binary: rdkit_desc + erg + avalon
